# A reciprocal inhibition model of alternations between under-/overemotional modulatory states in patients with PTSD

**DOI:** 10.1038/s41380-020-0827-0

**Published:** 2020-07-20

**Authors:** Toshinori Chiba, Kentaro Ide, Jessica E. Taylor, Shuken Boku, Hiroyuki Toda, Tetsufumi Kanazawa, Sumie Kato, Yuka Horiuchi, Akitoyo Hishimoto, Toru Maruyama, Taisuke Yamamoto, Miyako Shirakawa, Ichiro Sora, Mitsuo Kawato, Ai Koizumi

**Affiliations:** 1 The Department of Decoded Neurofeedback, Computational Neuroscience Laboratories, Advanced Telecommunications Research Institute International, Kyoto, Japan; 2 The Department of Psychiatry, Kobe University Graduate School of Medicine, Kobe, Japan; 3 The Department of Psychiatry, Self-Defense Forces Hanshin Hospital, Kawanishi, Japan; 4 Flower of Light Clinic for Mind and Body, Tokyo, Japan; 5 Department of Neuropsychiatry, Kumamoto University Faculty of Life Sciences, Kumamoto, Japan; 6 The Department of Psychiatry, National Defense Medical College, Tokorozawa, Japan; 7 The Department of Neuropsychiatry, Osaka Medical College, Osaka, Japan; 8 The Florey Institute of Neuroscience and Mental Health, Melbourne, VIC Australia; 9 Chiyoda-Shinryou Clinic, Tokyo, Japan; 10 Japan Ground Self-force, Test and Evaluation Command, Military Medicine Research Unit, Tokyo, Japan; 11 Yokohama City University Graduate School of Medicine, Kanagawa, Japan; 12 Self-Defense Forces Central Hospital, Tokyo, Japan; 13 Army Surgeon, Western Army Headquarters, Japan Ground Self-Defense Force, Kumamoto, Japan; 14 RIKEN Center for Advanced Intelligence Project (AIP), Tokyo, Japan; 15 Sony Computer Science Laboratories, Inc., Tokyo, Japan; 16 Japan Science and Technology Agency (JST), PRESTO, Tokyo, Japan; 17 Graduate School of Media and Governance, Keio University, Kanagawa, Japan

**Keywords:** Neuroscience, Psychiatric disorders

## Abstract

Patients with posttraumatic stress disorder (PTSD) appear to manifest two opposing tendencies in their attentional biases and symptoms. However, whether common neural mechanisms account for their opposing attentional biases and symptoms remains unknown. We here propose a model in which reciprocal inhibition between the amygdala and ventromedial prefrontal cortex (vmPFC) predicts synchronized alternations between emotional under- and overmodulatory states at the neural, behavioral, and symptom levels within the same patients. This reciprocal inhibition model predicts that when the amygdala is dominant, patients enter an emotional undermodulatory state where they show attentional bias toward threat and manifest re-experiencing symptoms. In contrast, when the vmPFC is dominant, patients are predicted to enter an emotional overmodulatory state where they show attentional bias away from threat and avoidance symptoms. To test the model, we performed a behavioral meta-analysis (total *N* = 491), analyses of own behavioral study (*N* = 20), and a neuroimaging meta-analysis (total *N* = 316). Supporting the model, we found the distributions of behavioral attentional measurements to be bimodal, suggesting alternations between the states within patients. Moreover, attentional bias toward threat was related to re-experiencing symptoms, whereas attentional bias away from threat was related with avoidance symptoms. We also found that the increase and decrease of activity in the left amygdala activity was related with re-experiencing and avoidance symptoms, respectively. Our model may help elucidate the neural mechanisms differentiating nondissociative and dissociative subtypes of PTSD, which usually show differential emotional modulatory levels. It may thus provide a new venue for therapies targeting each subtype.

## Introduction

Posttraumatic stress disorder (PTSD) is a debilitating disorder that develops after experiencing life-threatening traumatic events. It is a paradoxical disorder because individuals with PTSD can display seemingly opposing symptoms. Specifically, they may display re-experiencing symptoms where they automatically engage with traumatic cues but they may also display avoidance and dissociative symptoms where they actively stay away from such cues [1, 2]. Patients with strong dissociative symptoms are categorized as having the dissociative PTSD subtype, which was added to the fifth edition of the Diagnostic and Statistical Manual of Mental Disorders (DSM-V) [1]. These participants exhibit multiple characteristics, including lower treatment response and a higher suicidal risk [3], which are distinct from those exhibited by typical PTSD patients, who are referred to as the “nondissociative subtype.”

In the previously proposed “model of emotional under- and overmodulation in PTSD,” patients with the nondissociative and dissociative PTSD subtypes were postulated to differ in their emotional modulation levels [2, 4]. In this model [2, 4], the nondissociative subtype was postulated to be characterized by “emotional undermodulation.” Emotional undermodulation is related to hyperarousal (re-experiencing and hypervigilance) symptoms and was postulated to be driven by failure of the ventromedial prefrontal cortex (vmPFC) to adequately inhibit the amygdala [2, 5]. Conversely, the dissociative subtype was postulated to be characterized by “emotional overmodulation.” Emotional overmodulation is related to hypoarousal (dissociation) symptoms and was postulated to be driven by over-inhibition of the amygdala by the vmPFC [1, 2].

Emotional modulatory levels of PTSD patients appear to manifest not only in their clinical symptoms but also in their attentional biases to threat. A healthy level of attentional bias toward threat allows one to detect imminent threats rapidly and to adaptively avoid them [6, 7]. While bias toward threat is found to be overemphasized in most anxiety disorders, as well as in PTSD [8, 9], some patients with PTSD have been reported to instead display attentional bias away from threat [10]. In traditional analyses of attentional bias, an overall bias toward threat is associated with characteristics of emotional undermodulation [9], while an overall bias away from threat has been implicated to be associated with characteristics of emotional overmodulation [11]. Previous results suggest that attentional biases may be unstable and fluctuate in patients with PTSD. This “attentional bias variability” is usually viewed as a reflection of “instability” in threat monitoring in patients with PTSD [12, 13].

Although there has been much progress in PTSD research, there remain two critical issues. First, although variabilities in emotional modulatory symptoms and in attentional biases have been shown to be associated, it remains unclear whether they share the same underlying neural mechanisms. A conceptual neural network model which assumes that these do share common neural mechanisms would provide a novel coherent framework to unify the different PTSD characteristics found at the neural, behavioral, and symptom levels. Second, it also remains to be examined how much emotional modulatory levels of individual PTSD patients can change over time. The current theoretical and clinical consensus is that PTSD is a dynamic disorder that involves fluctuations in emotional modulatory levels [1, 14–16]. Consistent with this, previous studies have reported fluctuations in behavioral, physiological, and neuroimaging responses within individual patients with PTSD [1, 14–16]. It remains unclear whether these previous results merely reflect stochastic noise causing random jitters in emotional modulatory level (e.g., jittering between slightly more and slightly less undermodulation). An alternate account might be that, if emotional under- and overmodulation are driven by common neural mechanisms, then previous results might reflect alternations between two opposing “emotional modulatory states” within the same PTSD patients. If such alternations were to emerge from common neural mechanisms then they should be found simultaneously at the neural activity, attentional bias, and clinical symptom levels. Given the strongly opposing features between emotional under- and overmodulation, the alternative account may better explain the PTSD characteristics.

In our “reciprocal inhibition model” we propose that PTSD patients alternate between emotional under- and overmodulatory states and that this occurs because the amygdala and vmPFC take turns inhibiting one another. Generally, reciprocally inhibiting circuits produce such rhythmic switching [17–19]. In reciprocal inhibition, a pair of distinct neural circuits alternately dominate each other via mechanisms such as postinhibitory rebound and spike frequency adaptation [19, 20]. For example, neurons in one circuit that are initially inhibited may escape from the inhibitory modulation when their intrinsic membrane properties allow them to cross the spike threshold, which then inhibits neurons in the initially inhibiting circuit. Thus, two competing neural circuits take turns to induce two alternative states.

We propose that, when in the emotional undermodulatory state, the amygdala is activated and this causes the vmPFC to be inhibited. As a consequence of this, we propose that patients become biased toward threat and that re-experiencing symptoms manifest. Furthermore, we propose that, when in the emotional overmodulatory state, the vmPFC is activated and this causes the amygdala to be inhibited. As a consequence of this, we propose that patients become biased away from threat and that dissociative symptoms manifest. The difference between nondissociative/dissociative subtypes is assumed to be continuous. For specific details on this model and for circumstantial evidence supporting it from the literature, see the “Proposal of the reciprocal inhibition model” section below.

To test the reciprocal inhibition model, we conducted a behavioral meta-analysis (16 patient populations, total *N* = 491) and a behavioral experiment with PTSD patients (*N* = 20). To test the neural-level assumptions of the model, we also performed a meta-analysis of previous findings to examine the association between amygdala reactivity and PTSD symptoms using data from 12 populations (total *N* = 316). Here, we analyzed reactivity in the amygdala but not that in the vmPFC due to incoherent definitions of the vmPFC in previous studies [21–23].

In summary, we propose that reciprocal inhibition occurs between the amygdala and the vmPFC, causing them to switch in dominance. This reciprocal inhibition model comprehensively explains alternations between neural activity, attentional bias, and PTSD symptoms within patients, while also providing one possible mechanism behind the emergence of PTSD subtypes. This model may be used to promote further understanding and better treatments of each PTSD subtype in future.

### Proposal of the reciprocal inhibition model

**Fig. 1:**
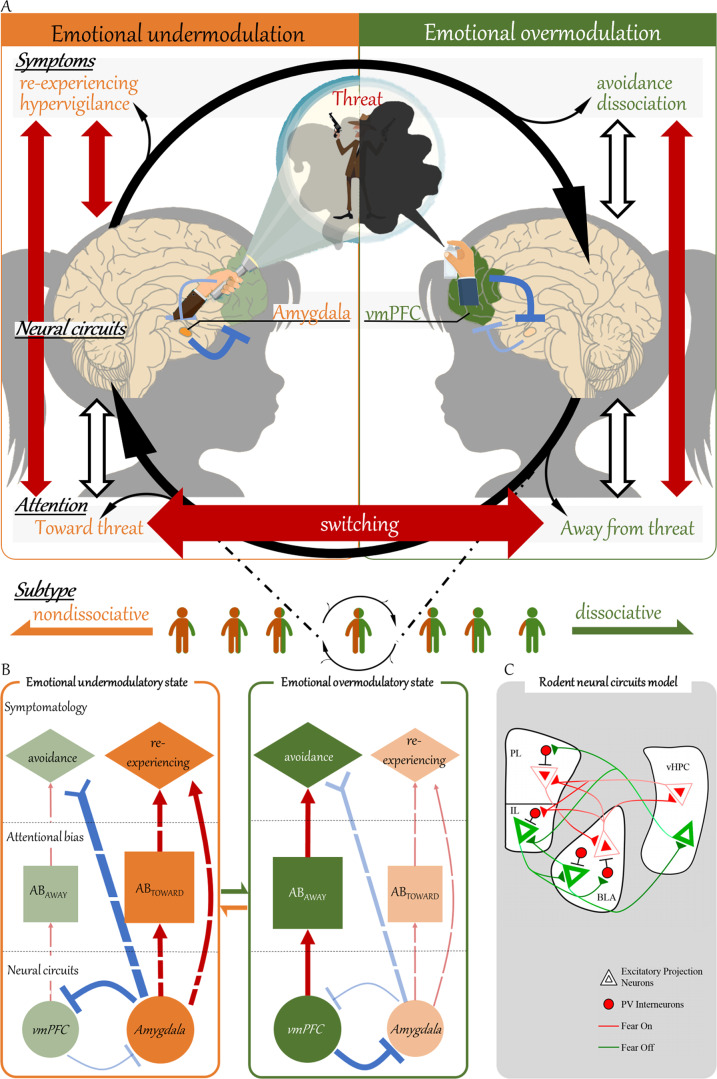
The reciprocal inhibition model, which explains switching between the emotional under- and overmodulatory states of PTSD. **a** In the reciprocal inhibition model, reciprocal inhibition between the amygdala and the vmPFC is proposed to contribute to alternations between the emotional under- and overmodulatory states in PTSD. Activation of the amygdala causes attention to be biased toward threat, subsequently causing re-experiencing symptoms to manifest (left-side). Conversely, activation of the vmPFC causes attention to be biased away from threat, subsequently causing avoidance/dissociative symptoms to manifest (right side). This model is an extension of “the model of emotional under- and overmodulation in PTSD” [4, 5]. Similar to this model [4, 5], the reciprocal inhibition model also predicts that the emotional modulatory states of a patient are determined by the degree to which the vmPFC regulates the amygdala. However, the reciprocal inhibition model further predicts that the amygdala can also act to modulate emotional state. This enables the reciprocal inhibition model to explicitly explain spontaneous switching between the two contrasting states. There is evidence in the literature supporting relationships represented by all vertical arrows on this figure, with the red (as opposed to white) vertical arrows representing evidence more directly supported by analyses in this paper. The red horizontal arrow indicates the switch between two states within a patient, which had not been shown before the results of this paper. Finally, the reciprocal inhibition model suggests that previously described (categorical) nondissociative and dissociative subtypes were determined depending on average measures of how long, and to what extent, individuals were on different points of this continuous measure of emotional regulatory state. **b** A putative generation of attentional bias and PTSD symptoms from amygdala/vmPFC activities in emotional under- and overmodulatory states. **c** The microcircuits that might underlie reciprocal inhibition. The amygdala (BLA “fear-on” neurons) and the vmPFC (IL “fear-off” neurons) reciprocally inhibit each other via activation of GABAergic inhibitory interneurons. PL prelimbic (medial prefrontal) cortex, IL infralimbic (medial prefrontal) cortex, vHPC ventral hippocampus, BLA basolateral amygdala, PV parvalbumin. Figure 1 (**c**) is adopted from Zimmermann et al. with permission from the authors [44].

We propose the reciprocal inhibition model to explain dynamic alternations of under- and overmodulatory states that reflect alternations in neural activity, attentional bias, and PTSD symptoms within patients. We hypothesize that reciprocal inhibition between the amygdala and vmPFC underlies the switching between emotional under- and overmodulatory states in PTSD (Fig. 1a, b). The emotional undermodulatory state is characterized by dominance of amygdala activity which results in suppressed vmPFC activity (Fig. 1a left). In this state, attentional bias is expected to be biased toward threat (AB_TOWARD_) so that patients automatically engage with traumatic cues and detect threatening stimuli faster. Furthermore, this is also expected to result in a prevalence of re-experiencing and hypervigilance symptoms, including greater fear responses toward threatening stimuli. Contrarily, the emotional overmodulatory state is characterized by dominance of vmPFC activity which results in suppressed amygdala activity (Fig. 1a right). In this state, attentional bias is expected to be biased away from threat (AB_AWAY_) so that patients detach themselves from traumatic cues and avoid perceiving threats. This is also expected to result in a prevalence of avoidance and dissociative symptoms, including reduced fear responses toward threatening stimuli.

The reciprocal inhibition model proposed in this paper is based on “the model of emotional under- and overmodulation in PTSD” [5]. We include three additional characteristics in the reciprocal inhibition model. First, not just the vmPFC but also the amygdala is hypothesized to act to modulate emotional state; this provides an explicit explanation for the neural mechanism behind spontaneous alternations between the two states. Second, in addition to variabilities in neural activities and clinical symptoms, our model incorporates behavioral level variability in attentional bias. Third, in our model individual differences in emotional modulation are treated as continuous, whereas previous studies treated them as categorical [1, 2]. Correspondingly, we suggest that the previously described (categorical) subtypes of PTSD were determined based on which inner emotional modulatory state the patient spent the most time in (Fig. 1a bottom).

The reciprocal inhibition model makes two critical predictions. First, the alternations between under- and overmodulatory states that arise from reciprocal inhibitory circuits should result in a bimodal distribution for behavioral measurements of attention (i.e., reaction times) for each individual patient. That is, when patients are in an emotional undermodulatory state, they are predicted to show attentional bias toward threatening stimuli. When patients are instead in an emotional overmodulatory state, they are predicted to show attentional bias away from threatening stimuli. Therefore, if reciprocal inhibition underlies attentional bias of patients with PTSD, then their reaction times for detection of threatening stimuli should show a bimodal distribution consisting of two peaks that arise from the emotional under- and overmodulatory states. Whether behavioral measurements of attention has a bimodal distribution is tested with our experimental data (*N* = 20). The second prediction is that if the same neural mechanism of reciprocal inhibition between the vmPFC and amygdala underlies both switches in attentional bias and in emotional modulatory state, then these should be found to alternate together in a predictable manner. This point is tested using the symptoms of re-experiencing and avoidance as proximates of under- and overmodulatory states. The difference between each patients’ re-experiencing and avoidance symptom scores, their “symptom imbalance,” was calculated as a proxy for the (im)balance between emotional under- and overmodulatory states for each patient and this was also used in analyses. Using behavioral meta-analysis, our experimental data, and neural meta-analysis, we tested whether the different emotional modulatory states, as reflected in our three measures of symptoms (i.e., re-experiencing, avoidance, and symptom imbalance), are distinctly associated with differences in attentional bias and neural reactivity of the amygdala.

The proposed model is circumstantially supported by the following empirical data. The amygdala and vmPFC appear to be generally reciprocally inhibitorily connected. A previous resting-state functional connectivity analysis of healthy individuals demonstrated that spontaneous fluctuations in the blood oxygen level-dependent (BOLD) signals of an amygdala subdivision were negatively correlated with those in the vmPFC [24]. Moreover, during processing of threat, the BOLD signal of the amygdala is negatively correlated with that of the vmPFC in healthy adults [25, 26]. In patients with PTSD, abnormality of the amygdala–vmPFC network is widely observed [27–35]. The amygdala–vmPFC network is characterized by a pattern of predominant bottom-up and top-down connectivity in the nondissociative and dissociative subtypes, respectively [36]. In addition, amygdala activity is positively correlated with attentional bias toward threat [37–39] and re-experiencing symptoms [9, 40, 41], while vmPFC activity is positively correlated with attentional bias away from threat [11, 42] and avoidance and/or dissociative symptoms [36, 43]. Findings from rodent studies suggest precise microcircuits that could, at least in part, underlie the reciprocal inhibition proposed by our model. In these studies, reciprocal inhibition is shown to occur between the amygdala “fear-on” neurons and vmPFC “fear-off” neurons via GABAergic inhibitory interneurons (Fig. 1c) [44–56].

In summary, there is a variety of circumstantial support for the existence of two “states,” in which either the amygdala or vmPFC activity is dominant, attention is either biased toward or away from threat, and either re-experiencing or avoidance symptoms are expressed (Fig. 1a, b).

### Classifications of PTSD symptoms in the reciprocal inhibition model

It is generally thought that, when possible, patients with PTSD try to prevent emotional engagement with trauma-related information as a strategy to actively avoid it [57], but otherwise they display emotional detachment symptoms as a strategy to dissociate themselves from the trauma. These different strategies have been theorized to be employed as coping mechanisms to deal with extreme distress in PTSD [2, 57]. These strategies also descriptively fit with the two symptom divisions of the avoidance cluster of the DSM-IV definition of PTSD: “active avoidance” and “emotional numbing.” In addition to emotional numbing, PTSD patients with the dissociative subtype sometimes show depersonalization and derealization as a form of emotional detachment. In general, active avoidance symptoms are not regarded as “dissociative,” whereas emotional numbing symptoms are [2, 5]. However, both (rodent behavior indicative of) active avoidance and emotional numbing symptoms, as well as other dissociative symptoms, have been shown related to increased vmPFC activity and reduced amygdala activity [2, 5]. Therefore, in this model “general” avoidance symptoms, including both active avoidance and emotional numbing, as well as other dissociative symptoms, are predicted to be dominant within the emotional overmodulatory state. Note that, although related, dissociative or nondissociative PTSD subtypes may therefore not directly correspond to emotional under- or overmodulatory states.

Given that re-experiencing and “general” avoidance symptoms are both necessary requirements to meet full PTSD criteria, measurements of these are available for all the diagnosed PTSD patients whose data were analyzed in this study. Therefore, we respectively used re-experiencing and “general” avoidance symptoms scores to index emotional under- and overmodulatory states for each of the patients. Furthermore, subtraction of re-experiencing from “general” avoidance symptoms, referred to here as “symptom imbalance,” allowed us to index the (im)balance between emotional under- and overmodulatory states for each of our patients.

## Methods

### Behavioral meta-analysis

#### Systematic literature search

In this behavioral meta-analysis, we extracted studies which assessed attentional bias using a dot-probe task, the most widely used procedure to measure attentional bias. We performed a systematic literature search using PubMed between October 1, 2019 and October 10, 2019. The following keywords were used in our search: “attentional bias” OR “attention bias,” AND “PTSD” OR “posttraumatic stress disorder” OR “acute stress disorder,” AND “dot-probe task.” The present systematic review follows the Preferred Reporting Items for Systematic Reviews and Meta-Analyses (PRISMA) guidelines. The inclusion criteria are presented in the PRISMA flow chart (Supplementary Fig. 1). We allowed behavioral studies to be included if they (1) included traumatized adults as participants; (2) were published in English; (3) reported PTSD subcluster scores; (4) reported reaction times in a dot-probe task, where the dot-probe replaced (a) trauma related compared to neutral stimuli, or, if these were not available, (b) general threat compared to neutral stimuli. It was decided that if the same individuals were tested multiple times and thus multiple symptom scores and attentional bias scores were reported, we would use the data from the test first taken after at least 1 month has passed since trauma exposure (so that PTSD studies would be prioritized over acute stress disorder studies). If studies reported the results of multiple dot-probe task procedures for the same individuals, we decided to only use the data from the more common procedure (see Supplementary Table 1 for details). Titles and abstracts were screened for eligibility by one assessor (TC; screening phase, *N* = 36). The full texts of all the finally included studies were examined in detail and independently selected by two assessors (KI, TC; *N* = 33). All the reference lists of the reviewed papers were examined to identify other eligible studies.

#### Statistical analysis of attentional bias and PTSD symptoms

For participants from each study selected for the behavioral meta-analysis, PTSD symptom imbalance was defined as the difference between the patients’ avoidance scores and their re-experiencing scores. Before subtraction, symptom scores were normalized so that the highest possible value was one and the lowest possible value was zero. Different studies measured their patients’ levels of PTSD using different versions of the DSM. The variables that contribute to avoidance scores in the DSM-IV protocol are divided into some that contribute to the avoidance scores and others that contribute to the emotional numbing scores in the DSM-V. Therefore, in studies where the DSM-V was used, the participants’ avoidance and emotional numbing scores were summed before being normalized to allow for direct comparisons between these studies and those that used the DSM-IV. Pearson correlation values were calculated between symptom imbalance and traditional attentional biases (TABs).

### Experiment

#### Participants

We enrolled 20 patients with PTSD (2 males, 18 females; mean age = 41.2 years; range = 22–53 years) from the Flower of Light Clinic for Mind and Body (*N* = 15) and the Chiyoda-Shinryou clinic (*N* = 2), which are both located in Tokyo, and the Shinchi-clinic (*N* = 3) located in Osaka. This sample size of 20 was predetermined based on previous PTSD studies on attentional bias variability [12, 58–60]. Using the DSM-IV, all the patients were diagnosed with PTSD resulting from domestic violence (*N* = 5), childhood abuse (*N* = 2), an unpleasant sexual experience (*N* = 2), or a combination of these (*N* = 11) according to the Clinician-Administered PTSD Scale (mean score = 80.6, SD = 20.2, range = 51–119, see Supplementary Table 2 for details). When considering that some participants experienced a combination (and so could be counted multiple times), 11 patients experienced domestic violence, 10 patients experienced an unpleasant sexual abuse, and 10 patients experienced childhood abuse. These participants reported strong fear when viewing pictures of angry male faces; were not taking psychotropic medication; had not suffered traumatic brain injury or loss of consciousness; and did not have any lifetime history of psychosis, alcohol abuse, or substance abuse. This study was approved by the Ethics Committees of the Central hospital of National Defense Force and Advanced Telecommunications Research Institute International (ATR). All the participants provided written informed consent.

#### Tasks and procedures

**Fig. 2:**
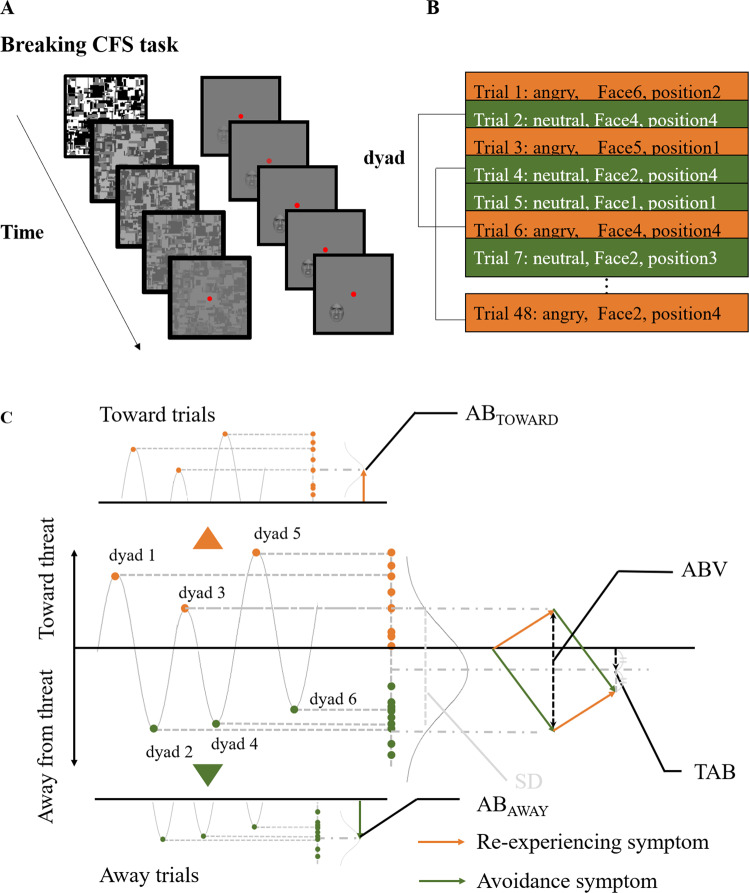
Examples of a breaking continuous flash suppression trial presentation and the putative generation of attentional bias during emotional under- and overmodulatory states. **a** An example of a breaking continuous flash suppression (b-CFS) trial presentation. During each trial, one of six different faces (Face1–Face6; angry or neutral expressions) was presented within one of four quadrants (position1 to position4) on a frame presented to one eye, while a Mondrian-like mask was simultaneously presented on a frame to the other eye. **b** Each dyad consisted of one neutral face and one angry face trial with consistent face identity (e.g., Face1) and position (e.g., position1). **c** For each dyad, the attentional bias score was defined as the difference between the reaction time to the angry face and that to the neutral face. Positive and negative values represent attentional bias toward and away from threat, respectively. We averaged attentional bias scores of all the dyads with positive and negative values separately and defined them as AB_TOWARD_ and AB_AWAY_. The standard deviations (SDs) of all the attentional bias scores in total (not split by valence) are comparable with attentional bias variability (ABV) measures used in previous studies. The averages of all the attentional bias scores in total are comparable with traditional attentional bias (TAB) measures used in previous studies. Since the biases toward and away from threat are indicative of re-experiencing and avoidance, respectively, we conceptually illustrated the putative generation of attentional bias during emotional under/overmodulatory states. That is, AB_TOWARD_ and AB_AWAY_ are associated with emotional under- and overmodulatory states, respectively.

In assessing the attentional bias scores, we used a breaking continuous flash suppression (b-CFS) method [61, 62] to overcome the methodological issues in conventional approaches. Conventional paradigms mainly adopt tasks involving conscious presentation of threat, which encourages participants to take widely different strategies [61]. Conventional procedures also adopt rather indirect approaches for measuring attentional biases, thereby making it difficult to differentiate the effect of attentional vigilance from participants’ difficulty in disengaging from threat [63]. The b-CFS is a promising alternative method because it can more directly measure subconscious processing of attentional bias for threat. The b-CFS task was adapted from the study by Yang et al. [61]. CFS renders a target stimulus invisible by presenting it to one eye while presenting a mosaic pattern to the other eye [64]. The b-CFS task assesses the detection time of stimuli masked by binocular suppression (Fig. 2a). Grayscale pictures of six male faces were obtained from the ATR Facial Expression Image database (DB99) and used as target stimuli. These pictures depicted angry or neutral expressions and were cropped in a circular shape to include the brows, eyes, nose, and mouth. All pictures were equated for contrast and luminance. Visual stimuli were presented using MATLAB (MathWorks, Inc.) with the Psychophysics Toolbox extensions [65, 66]. Stimuli were presented dichoptically through an Oculus Rift head-mounted display (Oculus, Inc.). To facilitate binocular fusion, one black “fusion frame” was displayed to each eye. A black fixation cross was drawn in the center of each fusion frame and the participants were instructed to remain fixated. Target stimuli were presented covering one of four quadrants within the fusion frames. Twelve pictures (6 males × 2 expressions) were presented once in every quadrant in a randomized order resulting in a total of 48 trials. To render stimuli invisible, achromatic Mondrian-like masks were flashed at 10 Hz to the dominant eye while target stimuli were presented to the other eye.

Each trial started with a fixation period of 4 s. Subsequently, suppression masks were flashed to the dominant eye, and the target stimulus gradually faded into the nondominant eye by linearly increasing its contrast over 1 s. During the 1–7 s period after trial onset, the contrast of the CFS masks was slowly decreased to zero. Stimuli were displayed until the participants pressed a key to indicate the quadrant in which the target stimulus (or any part of the target stimulus) emerged from suppression [61, 62]. The participants were required to respond as quickly as possible without compromising accuracy. The participants’ response cleared the screen.

To familiarize the participants with the procedure, they were presented with 12 practice trials (each of the 12 target stimuli was presented once) at the beginning of the experiment. Before beginning the experiment, eye dominance was examined using the hole-in-a-card test [67].

#### Three attentional bias scores

Reaction times for correct trials were the main outcome measured in this study. Trials with incorrect responses and one extreme outlier trial, in which the response time was more than five standard deviations above the participants’ mean for that particular condition, were excluded from subsequent analysis (<3% of all trials). As it stands in previous attentional bias studies, the variability within a patient is an important factor that is associated with PTSD symptomatology. In the reciprocal inhibition model, we assumed that outlier trials are important factors that determine variabilities. Therefore, we decided not to exclude any other outlier trials. Importantly, we ensured that excluding the one outlier trial that we did, did not compromise the results.

Overall, there was no difference in the percentage error rate between stimulus facial expressions (i.e., angry or neutral) (*t* = 1.228, df = 19, *p* = 0.23). During each trial, one of the six different faces (Face1 to Face6; angry or neutral expressions) was presented within one of four quadrants (position1 to position4) on a frame presented to one eye. The difference between the reaction times to the angry faces and the reaction times to the neutral faces was used to define overall TAB. We refined previously used attentional bias parameters for each participant [68] to generate a more stable index that was less influenced by the effects of face identity and the presented position. To this end, stimuli were paired in dyads consisting of one neutral face trial and one angry face trial with consistent face identity (e.g., Face1) and position (e.g., position1). Next, for each dyad, the TAB score was defined as the difference between the reaction time to the angry face and the reaction time to the neutral face. Since positive attentional bias scores represent attentional bias toward threat, we averaged all of the positive attentional bias scores and defined them as attentional bias score toward (AB_TOWARD_). Similarly, since negative attentional bias scores reflect attentional bias away from threat, we averaged all the negative attentional bias scores and defined them as AB_AWAY_. The absolute value of the participants’ AB_AWAY_ was used in subsequent analyses to allow easy comparisons of the magnitudes of attentional bias scores.

#### Statistical analysis of attentional bias scores and PTSD symptoms

A correlation analysis was performed between symptom imbalance (the difference between patients’ avoidance and re-experiencing scores) and TAB. Further, a stepwise multiple regression analysis was performed to evaluate the relationships between the three symptom clusters that are required to meet diagnostic criteria (re-experience, avoidance, and hypervigilance) and each attentional bias score (AB_TOWARD_ and AB_AWAY_). In this stepwise multiple regression, an automatic statistical model selection procedure was adopted as an exploratory means of identifying which symptom cluster/combination best explains the attentional bias scores. We confirmed that the current data met the assumption of multicollinearity, by computing the intercorrelation between the predictor variables (re-experience, avoidance, and hypervigilance) (see Multicollinearity in the data in Supplementary). In all analyses, *p* < 0.05 was considered statistically significant. In all correlation analyses in this paper, the *p* values reported are those that result from one-tailed tests. This is because we had strong a priori hypotheses about the direction of association between variables. All correlation analyses and regression analyses were performed as parametric tests. Since parametric analysis is not as robust for outlier effects as nonparametric analysis, we further ran nonparametric analyses to ensure that no significant results were driven by outliers. Specifically, analyses of variables that included outliers (exceeding two standard deviations) were also tested in nonparametric analyses. These nonparametric analyses showed qualitatively similar results to those derived from the parametric analyses (see Results of nonparametric analyses in Supplementary). In addition, we examined the assumption that both avoidance and dissociative symptoms (depersonalization/derealization symptoms) are emotional overmodulatory symptoms (see Classification of PTSD symptoms). Specifically, we performed analyses in which avoidance scores were substituted with the summation of avoidance and dissociative scores (see Analyses using the summation of avoidance symptoms and depersonalization/derealization scores in Supplementary).

#### Distributions of reaction times

After analyzing the association between attentional bias scores and PTSD symptoms, we analyzed the distributions of reaction times measured in the b-CFS task. First, we fitted Gaussian mixture models with one or two components to each participant’s reaction times as well as to overall reaction times collected from all the participants. Then, we compared the Akaike’s Information Criterion (AIC) of the models. Briefly, a smaller AIC means better fitting of the model. If there is an AIC difference greater than 2 between two models, then the model with the smaller AIC is regarded as the meaningfully better one. Here, trials with incorrect responses and one extreme outlier trial were excluded from the analyses as was done in calculation of attentional bias scores. All the detection times of a patient for angry and neutral faces were included in the analysis after normalizing the difference between mean reaction times to angry and neutral faces. After confirming a bimodal distribution, we then divided all the reaction times of a patient into two clusters using the k-means clustering algorithm. We performed a correlation analysis between the distance between the centers of each distribution cluster and the summation of re-experiencing and avoidance symptoms. Since the k-means clustering provided slightly different results based on randomly chosen initial values, we repeated the correlation analysis 100 times with 100 initial conditions. The average Pearson correlation value and the average *p* value are reported as well as their ranges in the “Results” section.

### Imaging meta-analysis

#### Systematic literature search

We performed a systematic literature search using PubMed between April 1, 2019 and April 20, 2019. The following keywords were used in our search: “fMRI” OR “functional magnetic resonance imaging” combined with AND “PTSD” OR “posttraumatic stress disorder” OR “acute stress disorder.” The present systematic review follows the PRISMA guidelines. The inclusion criteria are presented in the PRISMA flow chart (Supplementary Fig. 2). Neuroimaging studies were included if they (1) included traumatized adults as participants; (2) were published in English; (3) compared fMRI BOLD signals to (a) threatening stimuli vs. neutral stimuli, (b) stimuli that included threatening/unpleasant stimuli vs. stimuli that did not include threatening/unpleasant stimuli, or (c) threatening/unpleasant stimuli vs. baseline; and (4) reported (a) PTSD subcluster scores and (b) amygdala BOLD signals as *z*-scores or *t*-stats. Titles and abstracts were screened for eligibility by one assessor (TC; screening phase, *N* = 323). The full texts of all the finally included studies were examined in detail and independently selected by two assessors (KI, TC; *N* = 191). All the reference lists of the reviewed papers were examined to identify other eligible studies.

#### Statistical analysis of amygdala activity and PTSD symptoms

Symptom imbalance was defined in the same way as it was in the behavioral meta-analysis. Pearson correlation values were calculated between symptom imbalance and *z*-scores that represented left and right amygdala activity separately. Activities from the left and right amygdala were analyzed separately, considering some functional differences of the amygdala between the hemispheres [23, 69]. If a study included in the imaging meta-analysis did not provide a *z*-score, the *t*-stat was transformed into a *z*-score using the SPM12 built-in-function.

## Results

### Behavioral meta-analysis

#### Relationship between traditional attentional bias and symptom imbalance

**Fig. 3:**
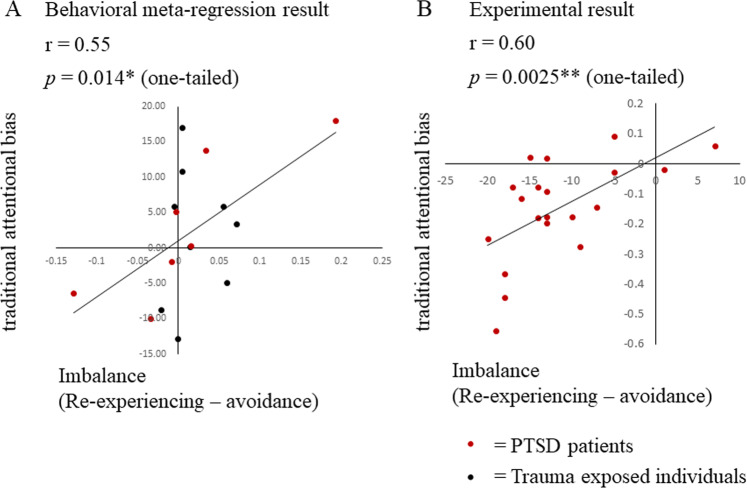
PTSD symptom imbalance and traditional attentional bias: results of the analyses of the behavioral meta-data and the data from our experiment. Each dot on the left graph showing the behavioral meta-analysis results represents the mean values, each point for one participant population, from systematically selected studies (Supplementary Fig. 1, Supplementary Table 1). In both graphs, the *x* axes denote the PTSD symptom imbalance, which was defined as the difference between the normalized avoidance and re-experiencing scores. The *y* axes denote traditional attentional bias to threat. Traditional attentional bias was assessed with dot-probe tasks in the meta-data (**a**) and with b-CFS in our experimental data set (**b**). In both analyses, positive values indicate attentional bias toward threat and negative values indicate attentional bias away from threat. Traditional attentional bias was positively correlated with symptom imbalance both in the meta-analysis and in our experimental results (meta-analysis: *r* = 0.55, *p* = 0.014, one-tailed, our experiment: *r* = 0.60, *p* = 0.0025, one-tailed). The meta-analysis includes trauma exposed individuals both with and without PTSD. Re-analysis of this data, with only the PTSD patients included, showed a stronger correlation between traditional attentional bias and symptom imbalance (*r* = 0.83, *p* = 0.011, one-tailed).

In the behavioral meta-analysis, the relationship between symptom imbalance and TAB was examined. The data from 16 participant populations (total *N* = 491) were extracted from 9 studies (see Supplementary Table 1 for details). Symptom imbalance was found to positively correlate with TAB (*r* = 0.55, *p* = 0.014, one-tailed: Fig. 3a). This indicates that a higher re-experiencing score predicted an attentional bias toward threat, whereas a higher avoidance score predicted an attentional bias away from threat.

### Experiment

#### Relationship between attentional bias and symptom imbalance

The b-CFS task was adapted to assess the attentional biases of 20 patients with PTSD in our experiment. Consistent with the results of the behavioral meta-analysis, symptom imbalance was positively correlated with TAB (*r* = 0.60, *p* = 0.0025, one-tailed: Fig. 3b). This indicates that a higher re-experiencing score is associated with rapid detection of threat, whereas a higher avoidance score is associated with delayed detection of threat. The attentional biases were then divided into attentional bias toward threat (AB_TOWARD_) and attentional bias away from threat (AB_AWAY_). The percentage of “toward trials” (Fig. 2c) was 46.3% (range 39.1–58.3%), which indicates that all the patients alternated between toward and away trials and is consistent with a previous study that showed alternations between the two attentional states [68].

**Fig. 4:**
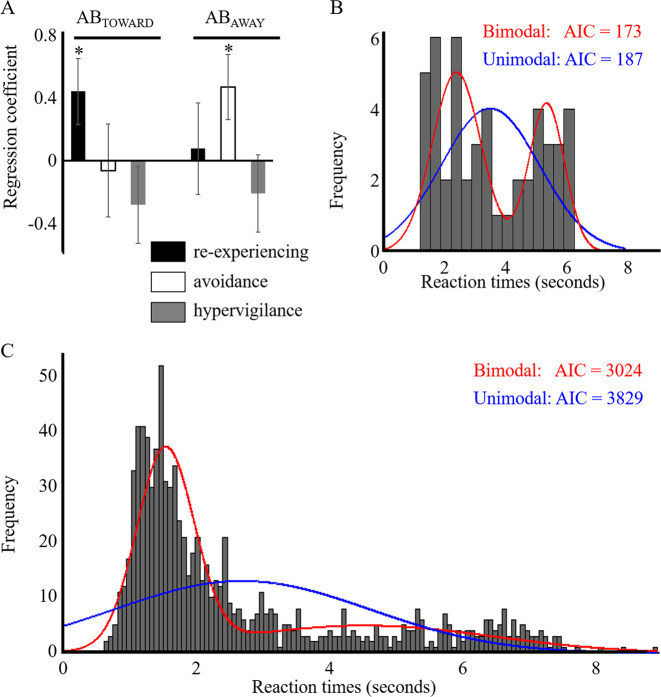
PTSD symptom clusters and attentional bias scores: results of the stepwise regression analysis and the distributions of reaction times to angry and neutral faces. **a** AB_TOWARD_ was associated with re-experiencing, and AB_AWAY_ was associated with avoidance. *p < 0.05. Distributions of reaction times to angry and neutral faces from the b-CFS task are shown for (**b**) one example patient and (**c**) all the patients. In both (**b**) and (**c**), blue and red lines indicate the fit of unimodal and bimodal models, respectively. Model comparisons based on AIC demonstrated that the bimodal-distribution models were better fitted to the reaction-time distributions than the unimodal-distribution models. This suggests that there were two peaks in reaction-time distributions, which is consistent with the prediction of our reciprocal inhibition model that under- and overmodulatory states alternate within patients. The peak with shorter reaction times is presumed to arise due to attention under the undermodulatory state causing faster detection of faces, and the peak with longer reaction times is presumed to arise due to attention under the overmodulatory state causing slower detection of faces (see Supplementary Fig. 4 where bimodal distributions for reactions times to angry and neutral faces are shown separately).

The first stepwise regression analysis revealed that the re-experiencing symptom cluster, but not the avoidance symptom cluster, was a significant predictor of AB_TOWARD_ (overall model: *r*^2^ = 0.20, df = 1, 18, *p* = 0.0499; re-experiencing: *β* = 0.44, *p* = 0.0499; avoidance: *β* = −0.06, *p* = 0.84; Fig. 4a left). The second stepwise regression analysis revealed that, on the contrary, the avoidance symptom cluster, but not the re-experiencing cluster, was a significant predictor for AB_AWAY_ (overall model: *r*^2^ = 0.22, df = 1, 18, *p* = 0.036; re-experiencing: *β *= 0.08, *p* = 0.79; avoidance: *β* = 0.47, *p* = 0.036; Fig. 4a right). Hypervigilance was not selected as an effective predictor in either of these stepwise regression analyses (*β* = −0.28, *p* = 0.27 for predicting AB_toward_ and *β* = −0.21, *p* = 0.41 for predicting AB_away_). The order of variables added into this model was automatically determined by the algorithm and was based solely on the *t*-statistics of their estimated coefficients. A higher re-experiencing score predicted a greater AB_TOWARD_ (Supplementary Fig. 3A), whereas a higher avoidance score predicted a greater AB_AWAY_ (Supplementary Fig. 3B) (see Supplementary Table 2 for details).

Interestingly, a similar relationship was observed between the PTSD symptom clusters and the variability within each direction of attentional bias (AB_TOWARD_ and AB_AWAY_). That is, the re-experiencing cluster score was significantly correlated with the variability within AB_TOWARD_ (*r*^2^ = 0.31, df = 1, 18, *p* = 0.005, one-tailed), whereas the avoidance cluster score was significantly correlated with the variability within AB_AWAY_ (*r*^2^ = 0.37, df = 1, 18, *p* = 0.0022, one-tailed). Analyses where avoidance symptoms scores were replaced by the summation of avoidance symptoms and depersonalization/derealization scores showed the similar results (see Analyses using the summation of avoidance symptoms and depersonalization/derealization scores in Supplementary).

#### Bimodal distributions of reaction times

Distributions of reaction times from the b-CFS task were analyzed to examine the prediction that individual patients switch between the two distinct states. The distributions of reaction times from 19 out of 20 individual patients were better fitted by a bimodal than a unimodal distribution (Fig. 4b) (bimodal: mean AIC = 110, std = 25.6, unimodal: mean AIC = 135, std = 11.5). AIC differences were greater than 2 for all 19 of these patients, which is indicative of meaningful differences between the “goodness” of unimodal (worse) and bimodal (better) fits. The distribution of average reaction times across all patients was also better fitted by a bimodal than a unimodal distribution (Fig. 4c), (bimodal: AIC = 3024, unimodal: AIC = 3829). Of note, we confirmed that bimodal distributions did not result from a pair of unimodal distributions, one with reaction times to neutral faces and the other with reaction times to angry faces (see also Supplementary Fig. 4).

The reciprocal inhibition model assumes that both the shorter peak in the bimodal distribution of reaction times and re-experiencing symptoms arise under the undermodulatory state. In contrast, the model assumes that both the longer peak of reaction times and avoidance symptoms arise under the overmodulatory state. Based on these assumptions, the shorter peak of reaction times is expected to become even shorter along with more severe re-experiencing symptoms under a more excessive undermodulatory state, while the longer peak of reaction times becomes even longer along with more severe avoidance symptoms under a more excessive overmodulatory state. If the two emotional modulatory states were to alternate within the same patients, then the distance between the two peaks of reaction times is predicted to co-vary with the distance between re-experiencing and avoidance symptoms. To test this prediction, we examined whether the distance between the two peaks of reaction times correlates with the distance between re-experience and avoidance symptoms. Here, the distance between re-experience and avoidance symptoms was estimated as the summation of those symptoms. This is because, if the severity of re-experience symptoms reflects the degree of undermodulatory state and if the severity of avoidance symptoms reflects the degree of overmodulatory state, then the summation of the symptoms is expected to reflect how far apart the two states are. Supporting the prediction, the distance between the two peaks was found to correlate positively with the summation of re-experiencing and avoidance symptoms (mean *r* = 0.42 range [0.40–0.45], mean *p* = 0.032 range [0.023–0.040], one-tailed, for the results from the 100 iterated correlations).

### Imaging meta-analysis

#### Relationship between amygdala activity and symptom imbalance

**Fig. 5:**
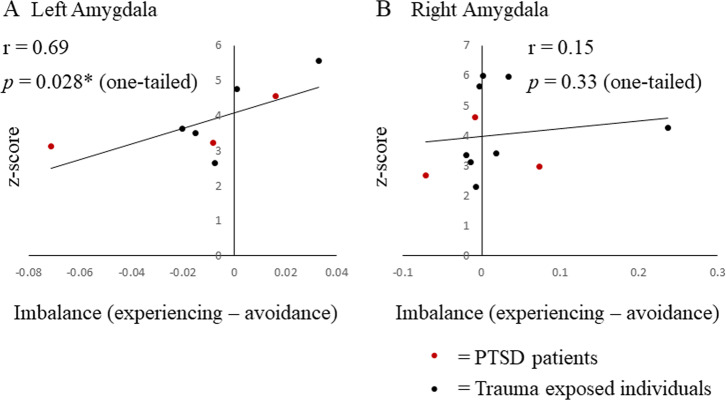
PTSD symptom imbalance and amygdala BOLD response: results of the imaging meta-analysis. Each dot represents the mean value for one participant population from systematically selected studies (Supplementary Fig. 2, Supplementary Table 3). The *x* axis denotes the PTSD symptom imbalance, which was defined the same way as in Fig. 3. The *y* axis denotes the fMRI BOLD signal to threat, expressed as *z*-scores. Left, but not right, amygdala BOLD signal was positively correlated with symptom imbalance (left amygdala: *r* = 0.69, *p* = 0.028, one-tailed, right amygdala: *r* = 0.15, *p* = 0.33, one-tailed).

In the imaging meta-analysis, the relationships between symptom imbalance and activity in left and right amygdala ROIs were examined. The data from 12 participant populations (total *N* = 316) were extracted from 9 studies (see Supplementary Table 3 for details). Among these, left amygdala activity was reported in 8 populations, while right amygdala activity was reported in 11 populations. We found results consistent with our prediction, as well as consistent with a previous report of greater relation between left, in comparison with right, amygdala activity and PTSD characteristics [23]. Specifically, we found symptom imbalance to be significantly correlated with left amygdala activity (*r* = 0.69, *p* = 0.028, one-tailed: Fig. 5a), but not with right amygdala activity (*r* = 0.15, *p* = 0.33, one-tailed: Fig. 5b). This indicates that a higher re-experiencing score predicted higher left amygdala activity in response to threat, whereas a higher avoidance score predicted lower left amygdala activity in response to threat.

## Discussion

Although studies have reported an array of clinical differences between PTSD patients with emotional undermodulation and those with emotional overmodulation [1, 4, 36], little is known about whether individual patients experience switching between the emotional under- and overmodulatory states. In this study, we tested our model in which reciprocal inhibition between the amygdala and vmPFC is predicted to generate alternations between these states within individual patients. Supporting the predictions of our model, our results indicated that two emotional modulatory states exist and that these can alternate even within the same individual patient. The results further supported the assumption of our model that reciprocal inhibition between the amygdala and the vmPFC underlies the alternations between these two emotional modulatory states.

The results of the behavioral meta-analysis and our behavioral experiment supported the prediction that two opposing emotional modulatory states—which encompass corresponding attentional biases and symptoms—exist. Across patients, re-experiencing and avoidance symptoms were found to be respectively associated with attentional bias toward (AB_TOWARD_) and away from (AB_AWAY_) threat. Because attentional bias and symptoms were found to alternate together in this predicted manner, this result suggests that emotional under- and overmodulation at the levels of behavior and symptomology share a common neural mechanism.

In addition, the results of our behavioral experiment showed that these opposing emotional modulatory states can alternate within the same individual patient. Specifically, bimodal distributions of detection times during the b-CFS task, indicative of two opposing emotional modulatory states, were found not only in patients as a group but also within individual patients. Moreover, the distances between the two clusters of detection times in the bimodal distributions were positively correlated with the strength of re-experiencing and avoidance symptoms across patients. This correlation suggests that the degree of alternations between the two modulatory states at the level of symptom is related to the degree of alternations at the level of attentional bias within patients.

The results of the imaging meta-analysis supported the predictions of our model regarding neural mechanisms. Consistent with the hypothesis that symptom alternating dynamics of PTSD are produced from reciprocal inhibition between the amygdala and vmPFC, the imbalance between re-experiencing and avoidance symptoms was found to be correlated with left amygdala activity to threat. When there were relatively more re-experiencing symptoms, the left amygdala tended to be more active; when there were relatively more avoidance symptoms the left amygdala tended to be less active. This fits with the model proposal that the amygdala dominates the vmPFC during the emotional undermodulatory state, while the vmPFC dominates the amygdala during the emotional overmodulatory state. However, future experiments where the vmPFC and the amygdala are simultaneously imaged are required to more directly examine this basic proposal of the reciprocal inhibition model.

Our results may help to enhance treatment response by predicting better timing of treatment based on the emotional modulatory states of individual patients. For example, previous studies showed that patients with the dissociative PTSD subtype, which is characterized by reduced amygdala reactivity, usually show a lower treatment response [70, 71]. On the other hand, exaggerated amygdala reactivity, which is a typical characteristic of the nondissociative subtype, has been shown to predict a poor response to exposure-based therapy [72]. These results suggest that excessively under- or overmodulatory states may hamper the treatment response. Based on such findings and our model, we can hypothesize that treatment response at a given time point could be predicted by the current imbalance between amygdala and vmPFC activity within individual patients. When predicting treatment response, it is important to consider the general characteristics of reciprocal inhibitory circuits which could induce a switch of states within the short time scale found here but also in the order of weeks [19]. This may explain the different periods of symptom fluctuations observed in previous studies [73]. Future studies based on the reciprocal inhibition model may lead to development of new therapies to maximize their effectiveness by targeting the period of a given emotional modulatory state.

The reciprocal inhibition model may also aid in the understanding of subtype development in PTSD. Imbalance in the time a person spends in emotional under- and overmodulatory states, at the early phase of PTSD development, is predicted to get exaggerated in the long term by Hebbian-like synaptic plasticity [74]. Even if amygdala activity is only temporarily dominant in the early phase of PTSD development (Fig. 1a bottom), this might result in long-term potentiation of inhibitory synapses from the amygdala to the vmPFC and long-term depression of inhibitory synapses from the vmPFC to the amygdala. For people for whom this is the case, the undermodulatory state may progressively become more dominant, contributing to them to eventually receive the diagnosis of nondissociative PTSD. Along a similar vein, dominance of the vmPFC in early phases of PTSD development may eventually lead to a prolonged overmodulatory state and thus the eventual diagnosis of dissociative PTSD. Based on this synaptic plasticity reasoning, the amount of time a patient spends in each state may reflect how far along their disorder has progressed. In this way, the reciprocal inhibition model may partly explain the development of the PTSD subtypes.

Although our model may partially explain the development of PTSD subtypes, we note that emotional under- or overmodulatory states may not completely and directly correspond to current definitions of nondissociative and dissociative PTSD subtypes. This is because, in our model, we used avoidance symptoms as an index of the emotional overmodulatory state, considering that these are a prerequisite for PTSD diagnosis, and thus all patients express some avoidance symptoms regardless of subtype. Nonetheless, analyses of our experimental data showed the similar results regardless of whether the avoidance symptom cluster was used by itself or together with dissociative symptoms. While this suggests that common underlying neural mechanisms may underlie avoidance symptoms and dissociative symptoms, future analyses are required to fully understand relationships of the two states, symptoms, and subtypes.

Our results suggest that time spent in each emotional modulatory state differs between PTSD subtypes. That is, the findings of our behavioral experiment revealed that the relative frequency of the emotional undermodulatory state, as indexed by the number of trials on which attention was biased toward threat, was numerically but not statistically greater in patients with the nondissociative subtype compared with those with the dissociative subtype (*t* (19) = 1.38, *p* = 0.18). This result is consistent with the assumption of the inhibitory reciprocal inhibition model that the two emotional modulatory states alternate in a continuous manner. Future studies may further test this assumption with a larger sample size. This is because a medium effect size (Hedge’s *d* = 0.60) was found, which indicates that a sample size of *N* = 44 for each group is required to reach statistical significance.

Some limitations of this study are as follows. First, there was nonuniformity in the studies included in the behavioral meta-analysis, as well as those included in the imaging meta-analysis, regarding several factors such as the experimental conditions and preprocessing methods. However, despite these methodology differences, we still observed symptom imbalance to strongly correlate with both TAB and amygdala activity, which indicates the robustness of our findings. Second, we only selected nine studies each for the behavioral and imaging meta-analyses. However, each study reported the results with relatively large sample sizes (total *N* = 316, *N* = 491, respectively). Third, although neural evidence from previous studies, such as data from functional magnetic resonance imaging (fMRI) studies, may support individual fluctuations and a pivotal role of vmPFC, we did not obtain neuroimaging data in the current experimental study. Therefore, there is a need for future neuroimaging studies on patients with PTSD focusing on within individual alternating dynamics.

Although more extensive examination of our reciprocal inhibition model is necessary, we believe that the proposed model provides a novel useful tool for advancing the diagnosis and treatment of PTSD. Classifying patients with PTSD with different symptoms into different subtypes has allowed more careful analysis of their differential responses to psychological trauma, which is expected to lead to a more sophisticated understanding of the neurobiology and treatment of PTSD [5]. For example, understanding of the relationships between subtype classifications and related clinically important findings have been largely advanced by the “model of emotional under- and overmodulation in PTSD” [2, 5]. By extending their model to include alternating dynamics between the two different PTSD states within individual patients, our model should hopefully further such advancements.

Overall, our reciprocal inhibition model coherently explains the dynamic alternations and associations between PTSD neural states, attentional biases, and symptoms. Therefore, the reciprocal inhibition model may be useful as a unifying framework to understand the complicated alternating dynamics of the diverse characteristics of PTSD.

## Supplementary information

Supplemental material

## Data Availability

Supplementary information is available at MP’s website.
