# Supplementary material for: A reciprocal inhibition model of alternations between under-/overemotional modulatory states in patients with PTSD
Source: Mol Psychiatry. 2020 Jul 20;26(9):5023–39. doi: 10.1038/s41380-020-0827-0 (PMC8589665; doi:10.1038/s41380-020-0827-0)
Supplement: Supplementary file 1 — Supplemental material [file 41380_2020_827_MOESM1_ESM.docx]

**Supplementary Material**


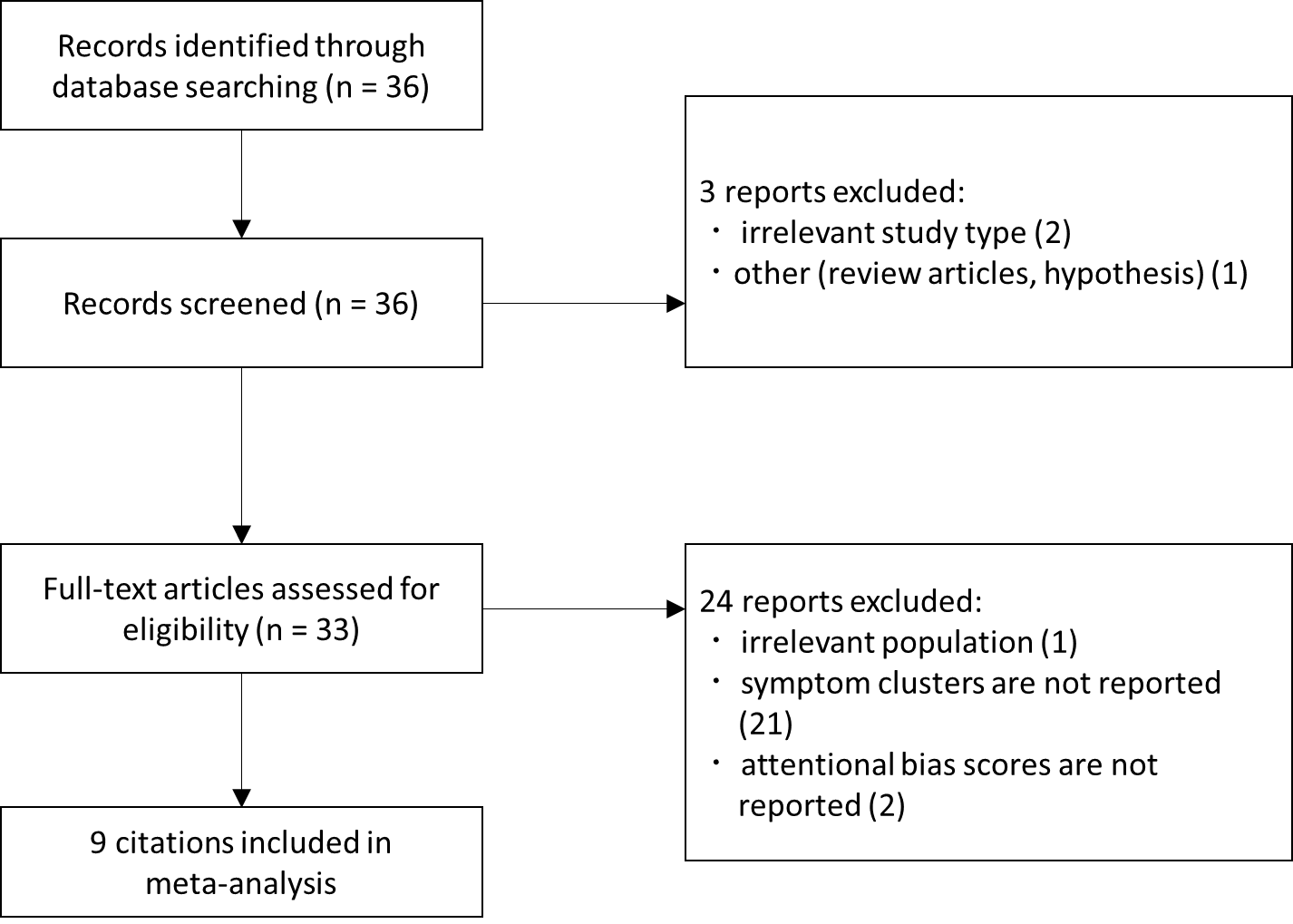


**Supplementary Figure 1. PRISMA chart for behavioral study selection.**

**
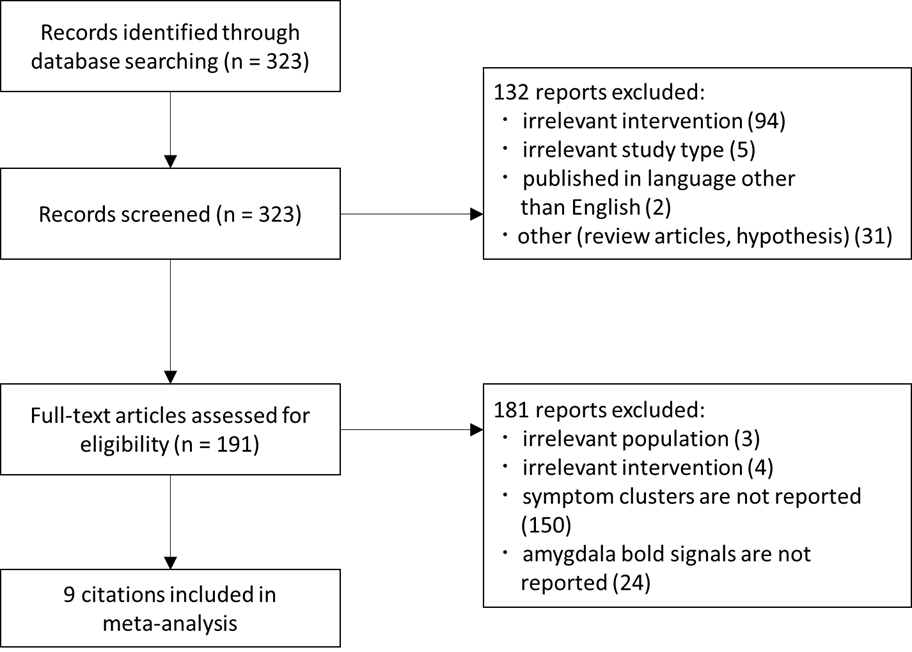
**

**Supplementary Figure 2. PRISMA chart for neuroimaging study selection.**


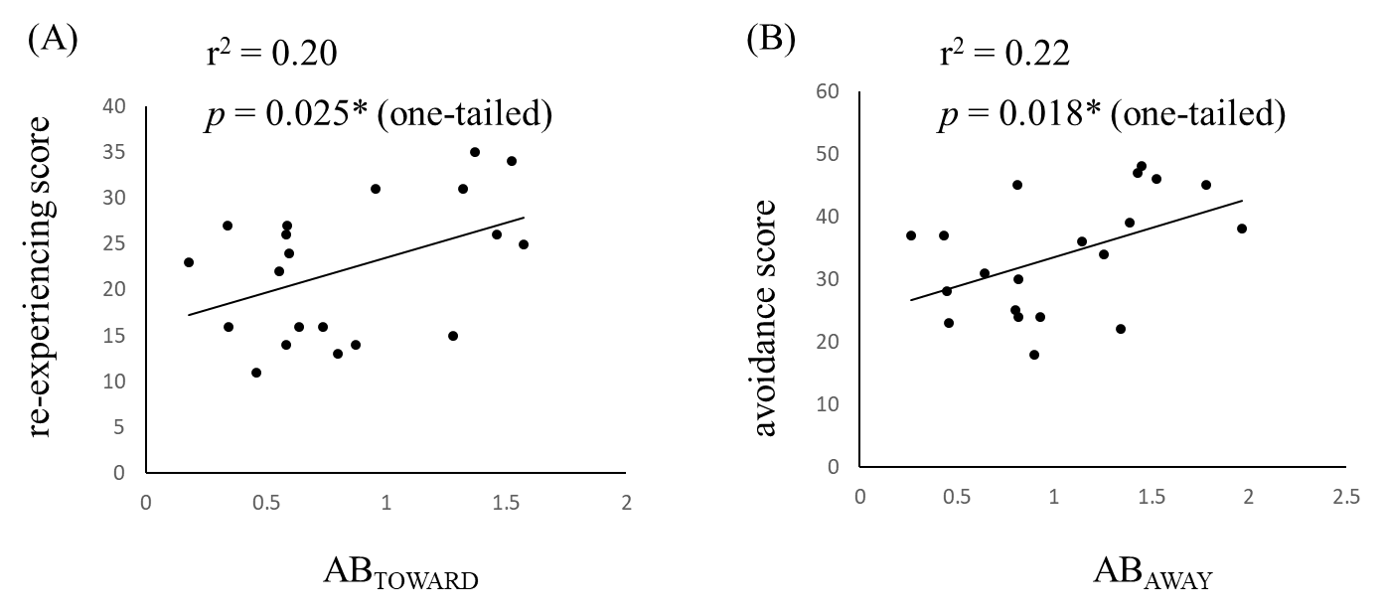


**Supplementary Figure 3. Relationships between PTSD symptoms and attentional biases.**

For demonstrative purposes, results of single-variable regression are shown here to visualize the relationships between (A) re-experiencing and AB_TOWARD_ and (B) avoidance and AB_AWAY_. * *p* < 0.05


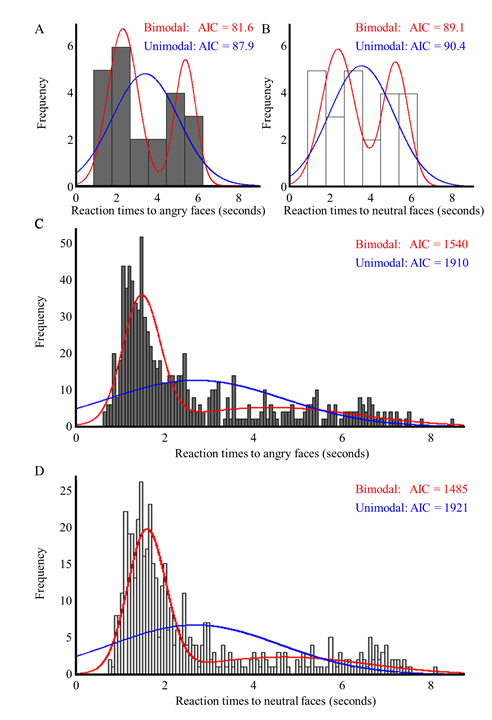


**Supplementary Fig. 4 The distributions of reaction times to angry faces and the distributions of those neutral faces**

Distributions of reaction times to faces from the b-CFS task are shown. Reaction times are shown to angry faces (A) and neutral faces (B) from one example patient. Reaction times are shown to angry faces (C) and neutral faces (D) from all the patients. In each panel, blue and red lines indicate the fit of unimodal and bimodal models, respectively. Model comparisons based on AIC demonstrated that the distributions of reaction times from nineteen out of twenty individual patients were better fitted with bimodal than unimodal distribution parameters, both for angry and for neutral faces (for angry faces bimodal: mean AIC =55.0, std = 3.4 and unimodal: mean AIC= 66.4, std =2.3; for neutral faces bimodal: mean AIC =50.7, std = 5.7 and unimodal: mean AIC= 59.6, std =6.2) (A & B). Of these, bimodal distribution models can be regarded as meaningfully better fit (AIC difference >2) than unimodal distribution models in seventeen patients for angry faces and in fifteen patients for neutral faces. The distributions of average reaction times across all patients were also better fitted with a bimodal than a unimodal distribution, both for angry (bimodal: AIC =1540, unimodal: AIC= 1910) and for neutral faces (bimodal: AIC =1485, unimodal: AIC= 1921) (C& D). These results indicate that bimodal distributions did not result from pairs of unimodal distributions, one set with reaction times to neutral faces and the other set with reaction times to angry faces. Here, the finding that bimodal distributions of reaction times were observed not only with angry faces (A & C) but also with neutral faces (B & D) is in line with the reciprocal inhibition model, as it predicts dominance of amygdala activity (over vmPFC activity) during the undermodulatory state and dominance of vmPFC activity (over amygdala activity) during the overmodulatory state. It has been previously shown that the amygdala responds not only to angry faces [1] but also to neutral faces [2, 3]. Thus, during the bCFS task, it is expected that reaction times to neutral faces also co-vary with the alternations between two emotional modulatory states, resulting in a bimodal distribution.

**Supplementary Table 1. Summary of the characteristics of the studies included in the behavioral meta-analysis**

| **Author** | **Scale** | **Condition** | **Populations (N)** | **Imbalance** | **Re-experiencing*** | **Avoidance*** | **TAB**** |
| --- | --- | --- | --- | --- | --- | --- | --- |
| Cowden Hindash [4] | CAPS | angry/fearful > neutral face | Female PTSD (24) | 0.035 | 0.366 | 0.331 | 13.75 |
|  |  |  | Male PTSD (17) | -0.008 | 0.374 | 0.381 | -2.00 |
|  |  |  | Female TEI (14) | 0.000 | 0.034 | 0.034 | -12.76 |
|  |  |  | Male TEI (15) | -0.005 | 0.052 | 0.057 | 5.93 |
| Powers [5] | CAPS  *** | angry > neutral face | TEI (54) | 0.055 | 0.213 | 0.158 | 5.88 |
| Swick  [6] | PCL  *** | trauma related > emotionally neutral pictures | PTSD (28) | 0.015 | 0.565 | 0.550 | 0.20 |
|  |  |  | TEI (28) | 0.016 | 0.107 | 0.091 | 0.20 |
| Schoorl [7] | SRIP | general threat > emotionally neutral pictures | PTSD (99) | -0.033 | 0.600 | 0.633 | -10.00 |
| Depierro [8] | PCL | trauma related > emotionally neutral words | TEI (27) | 0.005 | 0.262 | 0.256 | 17.04 |
| Fani  [9] | PSS | angry > neutral face | PTSD (18) | -0.128 | 0.353 | 0.481 | -6.49 |
|  |  |  | TEI (19) | -0.021 | 0.093 | 0.114 | -8.67 |
| Bardeen [10]**** | PCL | general threat > emotionally neutral pictures | TEI (58) | 0.005 | 0.180 | 0.175 | 10.90 |
| Elsesser [11] | IES-R | trauma related > emotionally neutral pictures | PTSD+TEI (35) | -0.002 | 0.168 | 0.171 | 5.14 |
| Elsesser [12] | IES-R | angry/fearful > neutral face | PTSD (18) | 0.194 | 0.595 | 0.402 | 17.93 |
|  |  |  | ASD (17) | 0.060 | 0.383 | 0.324 | -4.88 |
|  |  |  | THC (20) | 0.071 | 0.240 | 0.169 | 3.46 |

TEI: trauma-exposed individuals, ASD: acute stress disorder, TAB: traditional attentional bias

*PTSD symptoms were normalized such that the highest possible value was one and the lowest possible value was zero.

**Attentional bias was calculated by subtracting trials where the probe replaced a threat image from trials where the probe replaced a neutral image. Negative attentional bias scores indicate greater attention to neutral stimuli and positive attentional bias indicate greater attention to threat stimuli.

***The PTSD scale was measured using the structured clinical interview for DSM-V (SCID). Avoidance and numbing symptoms were combined into one category to allow for direct comparison of studies that used the DSM-IV with those that used the DSM-V.

**** In this paper two attentional bias scores were calculated based: one for stimuli presented for 150ms and the other for stimuli presented for 500ms. Since all the other studies reported in our behavioral meta-analysis used attentional bias scores calculated from stimuli that were presented for 500ms or longer, from this paper we used only the scores to stimuli that were presented for 500mss.

**Supplementary Table 2. Demographic data of PTSD patients**

| **ID** | **Sex** | **Age** | **Trauma type** | **Re- experiencing** | **Avoidance** | **Hypervigilance** | **Dissociation** | **Dissociative subtype*** | **AB_TOWARD_** | **AB_AWAY_** |
| --- | --- | --- | --- | --- | --- | --- | --- | --- | --- | --- |
| 1 | Female | 38 | Unpleasant sexual experience | 16 | 36 | 20 | 0 | 0 | 0.64 | 1.14 |
| 2 | Female | 48 | Domestic violence | 11 | 28 | 23 | 0 | 0 | 0.46 | 0.45 |
| 3 | Female | 53 | Domestic violence, Childhood abuse, Unpleasant sexual experience | 27 | 46 | 23 | 0 | 0 | 0.59 | 1.52 |
| 4 | Female | 46 | Domestic violence, Childhood abuse, Unpleasant sexual experience | 35 | 48 | 36 | 0 | 0 | 1.37 | 1.45 |
| 5 | Female | 51 | Domestic violence | 31 | 45 | 34 | 0 | 0 | 1.32 | 1.78 |
| 6 | Female | 38 | Domestic violence, Unpleasant sexual experience | 13 | 18 | 20 | 0 | 0 | 0.80 | 0.90 |
| 7 | Male | 53 | Childhood abuse, Unpleasant sexual experience | 26 | 39 | 11 | 0 | 0 | 1.46 | 1.39 |
| 8 | Female | 35 | Childhood abuse, Unpleasant sexual experience | 15 | 22 | 17 | 2 | 0 | 1.28 | 1.34 |
| 9 | Female | 53 | Domestic violence | 16 | 34 | 31 | 0 | 0 | 0.73 | 1.25 |
| 10 | Female | 29 | Domestic violence, Childhood abuse | 24 | 23 | 26 | 0 | 0 | 0.59 | 0.46 |
| 11 | Female | 24 | Domestic violence, Childhood abuse | 22 | 37 | 27 | 3 | 1 | 0.55 | 0.43 |
| 12 | Female | 31 | Domestic violence, Unpleasant sexual experience | 14 | 30 | 14 | 8 | 1 | 0.58 | 0.81 |
| 13 | Female | 22 | Unpleasant sexual experience | 14 | 24 | 25 | 6 | 1 | 0.87 | 0.93 |
| 14 | Female | 41 | Domestic violence, Unpleasant sexual experience | 34 | 47 | 30 | 12 | 1 | 1.52 | 1.43 |
| 15 | Female | 49 | Childhood abuse, Unpleasant sexual experience | 23 | 37 | 26 | 3 | 1 | 0.18 | 0.26 |
| 16 | Female | 47 | Domestic violence | 16 | 25 | 18 | 5 | 1 | 0.34 | 0.80 |
| 17 | Male | 29 | Childhood abuse | 26 | 31 | 25 | 4 | 1 | 0.58 | 0.64 |
| 18 | Female | 46 | Domestic violence, Childhood abuse, Unpleasant sexual experience | 25 | 38 | 24 | 3 | 1 | 1.57 | 1.97 |
| 19 | Female | 38 | Childhood abuse | 27 | 45 | 36 | 8 | 1 | 0.34 | 0.81 |
| 20 | Female | 53 | Domestic violence | 31 | 24 | 23 | 7 | 1 | 0.95 | 0.81 |
| Mean | - | 41.2 | - | 22.3 | 33.85 | 24.45 | 3.05 | 0.5 | 0.84 | 1.03 |

Symptoms are measured by the Clinician-Administered PTSD Scale for DSM–4.

*In order to meet the criteria for the dissociative subtype, individuals must additionally report as having depersonalization and/or derealization. We used the “1-2 rule” to define the presence of these symptoms, counting a symptom as present if it occurred at least monthly with at least moderate intensity.

**Supplementary Table 3. Summary of the characteristics of the studies included in the neuroimaging meta-analysis**

| **Author** | **Scale** | **Condition** | **Populations (N)** | **Imbalance** | **Re-experiencing*** | **Avoidance*** | **ltAmyg**** | | **rtAmy**** | |
| --- | --- | --- | --- | --- | --- | --- | --- | --- | --- | --- |
|  |  |  |  |  |  |  | **z-score** | **MNI xyz** | **z-score** | **MNI xyz** |
| Naegeli  [13] | CAPS | white noise burst > baseline | PTSD (28) | 0.016 | 0.463 | 0.446 | 4.54 | -20  4  -24 | N.S. | - |
| Lieberman [14] | SCID  *** | happy, angry, fearful, sad faces > shapes | TEI (45) | 0.033 | 0.298 | 0.265 | 5.57 | -28  -6  -18 | 5.98 | 20 -8 18 |
| Stevens  [15] | PSS | fear > neutral face | PTSD+  TEI (31) | 0.018 | 0.313 | 0.295 | N.S. | - | 3.44 | 46 0  -30 |
| Frijling [16] | CAPS | fear > happy face | ASD (41) | 0.237 | 0.423 | 0.186 | N.S. | - | 4.28 | 28 -7 -17 |
| Rabinak  [17] | CAPS | unpleasant > neutral images | PTSD (21) | -0.008 | 0.420 | 0.429 | 3.23 | -24  -8  -16 | 4.60 | 26  -2  -20 |
|  |  |  | TEI (21) | -0.020 | 0.010 | 0.030 | 3.65 | -20  -8  -16 | 3.37 | 24  -4  -14 |
| Stevens  ****[18] | PSS | fear > neutral face | PTSD+  TEI (22) | -0.015 | 0.247 | 0.262 | 3.52 | -32  -8  -16 | 3.12 | 24  -4  -28 |
| vanRooij [19] | CAPS | unpleasant > neutral images | TEI (28) | 0.001 | 0.018 | 0.017 | 4.76 | -20  -4  -20 | 6.00 | 20  -4  -16 |
|  |  |  | HC (25) | -0.004 | 0.017 | 0.021 | N.S. | - | 5.65 | 24  -4  -16 |
| Aupperle [20] | CAPS | anticipation of unpleasant > neutral images | PTSD (14) | 0.073 | 0.520 | 0.446 | N.S. | - | 2.96 | 20  -14  -10 |
| Stevens  **** [21] | PSS | fear > neutral face | PTSD (20) | -0.071 | 0.467 | 0.538 | 3.13 | -32  -4  -20 | 2.69 | 40  -4  -28 |
|  |  |  | TEI (20) | -0.008 | 0.107 | 0.114 | 2.65 | -28  -12  -12 | 2.31 | 24  -4  -28 |

TEI: trauma-exposed individuals, ASD: acute stress disorder, HC: healthy control, MNI: Montreal Neurological Institute

*PTSD symptoms were normalized such that the highest possible value was one and the lowest possible value was zero.

**Amygdala activations are described as z-scores.

***The PTSD scale was measured using the structured clinical interview for DSM-V (SCID). Avoidance and numbing symptoms were combined into one category to allow for direct comparison of studies that used the DSM-IV with those that used the DSM-V.

****The subsets of populations from these two studies are partially overlapping.

**Multicollinearity in the data**

To test the multicollinearity in the variables used in the step-wise multiple regression, the relationships between attentional bias scores and the three symptom clusters (re-experiencing, avoidance, and hypervigilance) were examined. We did not find multicollinearity in the data. The coefficients for the correlations between re-experiencing and avoidance (r = 0.69), avoidance and hypervigilance (r = 0.53), and re-experiencing and hypervigilance (r = 0.53) were below the suggested cut-off value of 0.8 [22].

**Results of non-parametric analyses**

To ensure that no significant results were driven by outliers, analyses of variables that included outliers (exceeding 2 standard deviations) were also tested with non-parametric analyses. In total, four correlational analyses were tested in non-parametric analyses. These non-parametric analyses showed qualitatively similar results to those derived from the parametric analyses

Specifically, traditional attentional bias was positively correlated with symptom imbalance both in the meta-analysis and in our experimental results (meta-analysis: Spearman’s rho = 0.53, *p* = 0.017, one-tailed, our experiment: Spearman’s rho = 0.59, *p* = 0.0032, one-tailed). In the neuroimaging meta-analysis, left, but not right, amygdala BOLD signal was positively correlated with symptom imbalance (left amygdala: Spearman’s rho = 0.64, p = 0.048, one-tailed, right amygdala: Spearman’s rho = 0.37, *p* = 0.13, one-tailed).

**Analyses using the summation of avoidance symptoms and depersonalization/derealization scores**

Analyses where the summation of avoidance symptoms and depersonalization/derealization scores were used showed similar results to analyses from the main text where just avoidance symptom scores were used. That is, even when the summation of avoidance symptoms and depersonalization/derealization scores were subtracted from re-experiencing symptom scores to calculate symptom imbalance, this was positively correlated with traditional attentional bias (r = 0.56, *p* = 0.0052, one-tailed). A stepwise regression analysis revealed that the re-experiencing symptom cluster was a significant predictor of AB_TOWARD_ (overall model: r^2^ = 0.20, df = 1, 18, *p* = 0.0499; re-experiencing: beta = 0.44), whereas the newly calculated avoidance + depersonalization/derealization symptom cluster was a marginally significant predictor for AB_AWAY_ (overall model: r^2^ = 0.15, df = 1,18, *p* = 0.093; avoidance: beta = 0.39).

**References**

1. Hariri AR, Tessitore A, Mattay VS, Fera F, Weinberger DR. The amygdala response to emotional stimuli: a comparison of faces and scenes. Neuroimage. 2002;17:317–323.

2. Young LR, Yu W, Holloway M, Rodgers BN, Chapman SB, Krawczyk DC. Amygdala activation as a marker for selective attention toward neutral faces in a chronic traumatic brain injury population. Neuropsychologia. 2017;104:214–222.

3. Wright P, Liu Y. Neutral faces activate the amygdala during identity matching. Neuroimage. 2006;29:628–636.

4. Cowden Hindash AH, Lujan C, Howard M, O’Donovan A, Richards A, Neylan TC, et al. Gender Differences in Threat Biases: Trauma Type Matters in Posttraumatic Stress Disorder. J Trauma Stress. 2019;32:701–711.

5. Powers A, Fani N, Murphy L, Briscione M, Bradley B, Tone EB, et al. Attention bias toward threatening faces in women with PTSD: eye tracking correlates by symptom cluster. Eur J Psychotraumatol. 2019;10:1568133.

6. Swick D, Ashley V. Enhanced Attentional Bias Variability in Post-Traumatic Stress Disorder and its Relationship to More General Impairments in Cognitive Control. Sci Rep. 2017;7:14559.

7. Schoorl M, Putman P, Van Der Werff S, Van Der Does AJW. Attentional bias and attentional control in Posttraumatic Stress Disorder. J Anxiety Disord. 2014;28:203–210.

8. DePierro J, D’Andrea W, Pole N. Attention biases in female survivors of chronic interpersonal violence: relationship to trauma-related symptoms and physiology. Eur J Psychotraumatol. 2013;4:19135.

9. Fani N, Jovanovic T, Ely TD, Bradley B, Gutman D, Tone EB, et al. Neural correlates of attention bias to threat in post-traumatic stress disorder. Biol Psychol. 2012;90:134–142.

10. Bardeen JR, Orcutt HK. Attentional control as a moderator of the relationship between posttraumatic stress symptoms and attentional threat bias. J Anxiety Disord. 2011;25:1008–1018.

11. Elsesser K, Sartory G, Tackenberg A. Initial symptoms and reactions to trauma-related stimuli and the development of posttraumatic stress disorder. Depress Anxiety. 2005;21:61–70.

12. Elsesser K, Sartory G, Tackenberg A. Attention, Heart Rate, and Startle Response During Exposure to Trauma-Relevant Pictures: A Comparison of Recent Trauma Victims and Patients With Posttraumatic Stress Disorder. J Abnorm Psychol. 2004;113:289–301.

13. Naegeli C, Zeffiro T, Piccirelli M, Jaillard A, Weilenmann A, Hassanpour K, et al. Locus Coeruleus Activity Mediates Hyperresponsiveness in Posttraumatic Stress Disorder. Biol Psychiatry. 2018;83:254–262.

14. Lieberman L, Gorka SM, DiGangi JA, Frederick A, Phan KL. Impact of posttraumatic stress symptom dimensions on amygdala reactivity to emotional faces. Prog Neuropsychopharmacol Biol Psychiatry. 2017;79:401–407.

15. Stevens JS, Kim YJ, Galatzer-Levy IR, Reddy R, Ely TD, Nemeroff CB, et al. Amygdala Reactivity and Anterior Cingulate Habituation Predict Posttraumatic Stress Disorder Symptom Maintenance After Acute Civilian Trauma. Biol Psychiatry. 2017;81:1023–1029.

16. Frijling JL, van Zuiden M, Koch SBJ, Nawijn L, Veltman DJ, Olff M. Effects of intranasal oxytocin on amygdala reactivity to emotional faces in recently trauma-exposed individuals. Soc Cogn Affect Neurosci. 2016;11:327–336.

17. Rabinak CA, MacNamara A, Kennedy AE, Angstadt M, Stein MB, Liberzon I, et al. Focal and aberrant prefrontal engagement during emotion regulation in veterans with posttraumatic stress disorder. Depress Anxiety. 2014;31:851–861.

18. Stevens JS, Almli LM, Fani N, Gutman DA, Bradley B, Norrholm SD, et al. PACAP receptor gene polymorphism impacts fear responses in the amygdala and hippocampus. Proc Natl Acad Sci U S A. 2014;111:3158–3163.

19. Van Rooij SJH, Rademaker AR, Kennis M, Vink M, Kahn RS, Geuze E. Neural correlates of trauma-unrelated emotional processing in war veterans with PTSD. Psychol Med. 2015;45:575–587.

20. Aupperle RL, Allard CB, Simmons AN, Flagan T, Thorp SR, Norman SB, et al. Neural responses during emotional processing before and after cognitive trauma therapy for battered women. Psychiatry Res. 2013;214:48–55.

21. Stevens JS, Jovanovic T, Fani N, Ely TD, Glover EM, Bradley B, et al. Disrupted amygdala-prefrontal functional connectivity in civilian women with posttraumatic stress disorder. J Psychiatr Res. 2013;47:1469–1478.

22. Hensher DA, Rose JM, Greene WH. Applied Choice Analysis: A Primer. Cambridge University

Press; 2005.
